# Regeneration in distantly related species: common strategies and pathways

**DOI:** 10.1038/s41540-017-0042-z

**Published:** 2018-01-11

**Authors:** Maria Rita Fumagalli, Stefano Zapperi, Caterina A. M. La Porta

**Affiliations:** 1 Department of Physics, Center for Complexity and Biosystems, University of Milan, via Celoria 16, 20133 Milano, Italy; 2 Department of Environmental Science and Policy, Center for Complexity and Biosystems, University of Milan, via Celoria 26, 20133 Milano, Italy; 3 CNR—Consiglio Nazionale delle Ricerche, Istituto di Chimica della Materia Condensata e di Tecnologie per l’Energia, Via R. Cozzi 53, 20125 Milano, Italy; 4 Department of Applied Physics, Aalto University, P.O. Box 11100, FIN-00076 Aalto, Espoo Finland

## Abstract

While almost all animals are able to at least partially replace some lost parts, regeneration abilities vary considerably across species. Here we study gene expression patterns in distantly related species to investigate conserved regeneration strategies. To this end, we collect from the literature transcriptomic data obtained during the regeneration of three species (*Hydra magnipapillata, Schmidtea mediterranea,* and *Apostichopus japonicus*), and compare them with gene expression during regeneration in vertebrates and mammals. This allows us to identify a common set of differentially expressed genes and relevant shared pathways that are conserved across species during the early stage of the regeneration process. We also find a set of differentially expressed genes that in mammals are associated to the presence of macrophages and to the epithelial–mesenchymal transition. This suggests that features of the sophisticated wound healing strategy of mammals are already observable in earlier emerging metazoans.

## Evolution: Common genetic signature of animal regeneration

All animals capable of regenerating a lost body part, from an organ or a limb to the whole organism, use a common set of genes. This is the striking discovery of a team of researchers from the Center for Complexity and Biosystems of the University of Milan, led by Caterina La Porta. They analyzed the genetic activity in regenerating tissues from widely different species—from hydra to mice. They found that some of the genes active at the beginning of the regeneration process are the same in very different species, including mammals which have lost this function during evolution, except for the restoration of the liver. The discovery of this shared genetic signature is of great importance to understand how regeneration evolved and could be useful for future regeneration therapies.

## Introduction

Regeneration, defined as the replacement of damaged or lost parts following injury without scarring or loss of functionality, is a widespread phenomenon present in almost all Metazoa.^1–3^ Regenerative capabilities across Metazoa display, however, a dramatically large degree of variability, both in terms of functional and morphological recovery. Regeneration capabilities range from the replacement of organs, tissues, and limbs up to the complete regrowth of whole organisms from body fragments,^2,4–7^ and can vary during the life cycle and with age.^4,8–11^ Regeneration is closely related to fundamental processes, such as cell proliferation, migration, and remodeling, but its complete success is linked to a precise regulation of a number of molecular mechanisms, since uncontrolled or biased proliferation could lead to anomalous morphologies.^12,13^

Differences in the regenerative capabilities are observable, not only comparing closely related species, but even between tissues within the same individual.^3,14–16^ It has been shown that through evolution the development of an immune system is followed by a concomitant loss of regenerative capacity.^4,9^ In mammals, the immune system can optimize tissue defense and repair but these animals cannot regenerate amputated body parts and this capability is limited to post-injury regeneration.^17,18^ A good model of this phenomenon is liver regeneration after partial hepatectomy (PH), where the original mass of the liver is re-established in direct proportion to the amount of tissue removed.

The aim of the present study is to establish a common genetic signature for regeneration processes in different species that could help us elucidate why mammals lost this function during evolution. To this end, we select three well-known model organisms with high regeneration capacity representative of different phyla in Metazoa: Hydra (Cnidaria), Planaria (Platyhelminthes) and Sea Cucumber (Echinodermata). We thus considered the very complete assembled transcriptome of *Hydra magnipapillata*,^19^ *Schmidtea mediterranea*,^20^ and *Apostichopus japonicus*.^21^ These data sets have been used as references in previous regeneration studies. For each species, we have access to both the reference and the regenerating transcriptome, obtained in the same experiment as a function of time. In order to reveal conserved patterns during the regeneration process, we map the different transcriptomes on a common genome.

*H. magnipapillata* is a paradigm for animal regeneration, being able to reconstruct missing body structure without scarring whatever its age.^22^ *S. mediterranea* represents the main model organism for the group of planarians, bilaterally symmetric Platyhelminthes that can be easily found in fresh water.^3^ Their regenerative capacity has been largely studied and is known to involve a population of adult stem cells present throughout the organism.^3,20^ *A. japonicus* (sea cucumbers) are among the number of echinodermata possessing a good regenerative capacity. In particular, they can completely replace internal organs after evisceration, a mechanism also used as a defense strategy. We also consider axolotl (*Ambystoma mexicanum*) limb regeneration, as well as mouse and rat liver regeneration, after PH as illustrative of tissue regeneration in vertebrates and mammals.^23–25^

## Results

### Transcriptome annotation and comparison

In order to compare transcriptomes from different species, we have first to map them into a reference database that allows for a more reliable functional prediction. Hence, we align the genes of the regenerating species considered here (i.e. *H. magnipapillata*, *S. mediterranea*, *A. japonicus*) to the complete set of the Uniprot/Swissprot protein database.^26^ Swissprot sequences represent a well-established and reliable subset of proteins, allowing us to exclude from the analysis non-coding transcripts and not curated sequences. While the Swissprot database refers to proteins, in the following we consider the associated genes. To reduce the noise, we group similar sequences into clusters that meet a similarity threshold of 80% according to the CD-Hit algorithm (see Methods) and consider each cluster as a single gene. This procedure results in the annotation of a smaller number of genes when compared to the alignment obtained using less-stringent databases, such as the NCBI non-redundant (NR) protein database^27^ (see Supplementary Fig. 1)

Using a permissive *p*-value threshold of 10^−2^, we obtain 11,643 genes annotated to at least a single eukaryotic gene in *H. magnipapillata*, 13,346 in *S. mediterranea*, and 9119 in *A. japonicus* (see Methods and Supplementary data 1). We measure the fraction of genes of these three species that can be annotated on different species contained in the Swissprot database and find that half of the annotation are human or murine, following the intrinsic species distribution of the reference database (Supplementary Figure 2). Note that the trend is the same for all the three organisms considered. To annotate and compare genes on the Swissprot database, the natural choice is the human genome, since it allows for a a more reliable functional prediction with respect to other well studied model organisms, such as mouse or zebrafish. To this end we consider the first human gene (human best-match) in the list of possible annotations ranked by *p*-value (see Methods).

**Fig. 1:**
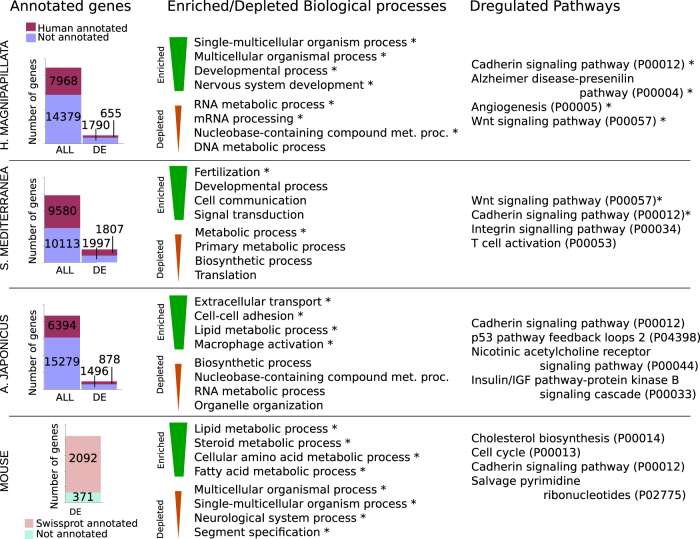
Transcriptome annotation. Figure shows the number of genes and DE genes annotated against at least one human gene according to Swissprot database for *H. magnipapillata*, *S. mediterranea*, and *A. japonicus*. Functional annotation of DE genes was performed for each species, top enriched and depleted Panther go-slim biological processes and pathways is shown. Swissprot annotation of DE genes during mouse liver regeneration is shown for comparison. Symbols indicate corrected *p*-value ≤ 0.05

Regeneration is a dynamic process involving a time-dependent gene expression.^20^ We thus analyze the regenerating transcriptomes (RTs) of *H. magnipalliata*,^19^ *S. mediterranea*^20^, and *A. japonicus*^21^ calculating, at each time point, the $\mathop {{{\rm log}}}\nolimits_2$ gene expression fold change (${\rm log_2}FC$) as compared to the initial condition. In this way, we obtain a list of differentially expressed (DE) genes (see Methods section). The resulting number of human annotated and DE genes for at least one time point during regeneration is reported in Fig. 1 for each of the three species separately. For comparison, we also show the number of DE genes during liver regeneration in mouse. Next, we perform functional classifications using Panther GO-slim annotation (see Methods section) and find a number of enriched and depleted biological processes and deregulated pathways. Direct comparison between the lists show some interesting observations, such as the common presence of metabolic processes across the species, as well as signaling pathways such as Wnt and cadherin (see Supplementary data 2).

### Early and late regeneration

In our datasets, three time points (6, 12, 24 h after injury) are shared among all the regeneration experiments except for *A. japonicus*, where the first time point is 3 days post intestine removal. Mouse liver regeneration data used in the previous section to investigate co-annotation include five time points up to the partial recovery of liver at 36 h post PH. Thus, here we consider only the dataset of DE genes after PH in rat^24^ consisting of a selection of 767 DE genes whose expression was measured over 10 different time points up to 7 days post injury, when the regrowth process can be considered complete. A simple solution to compare different timescales is to normalize time so that the final time points of the various regeneration processes coincide.

**Fig. 2:**
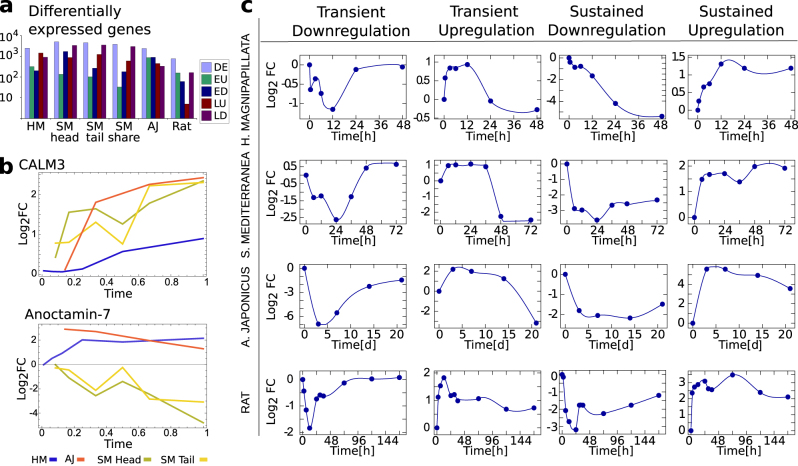
Pattern of expression of DE genes. **a** Total number of DE genes and early/late upregulated (EU/LU) and downregulated (ED/LD) genes for the considered organisms. The expression profile of *S. mediterranea* head and tail regenerations is considered separately, as well as the DE genes shared between the two processes. **b** The expression profile of two illustrative human genes DE in all the considered regeneration processes. Calmodulin show a coherent expression profile among organisms, while Anoctamin-7 is differentially expressed in the organisms. Time is rescaled and normalized in order to make the timescales comparable. **c** Figure shows the average expression level as a function of time of exemplificative clusters of genes resulting transiently down/upregulated and continuously down/upregulated during the regeneration process for *H. magnipapillata*, *S. mediterranea*, *A. japonicus*, and rat

Once the time points have been properly normalized, we can define an early and a late regeneration phase for each species. Figure 2a reports the number of DE genes for these two phases in all the species considered and shows a similar number in all cases, even when considering different body parts of the same species (*S. mediterranea*). Using rescaled time, we compare the change of expression of a subset of the human best-match co-annotated genes. Some genes have expression patterns that are comparable among all the species, while others are dramatically different (see Fig. 2b for two examples). Next, for each organism, putative DE genes are clustered according to their global expression profile during the regeneration process. This allows us to identify four recurrent expression patterns: genes that are (i) upregulated or (ii) downregulated in the early phase and that return to their basal expression level during the late phase, and (iii) genes that are constantly downregulated or (iv) upregulated over time (Fig. 2c). From the illustrative expression patterns reported in Fig. 2c, we can observe that in cases (i) and (ii) the maximum deviation from the basal expression level occurs $\approx 12\,{\rm h}$ after injury. Thus, we define as transiently early upregulated (EU) or early downregulated (ED) those genes having a significant increase or decrease in the first two time points after injury, and almost recover a basal expression after 24 h. For *A. japonicus* we considered a recovery time 14 days post injury.

The distinction between early and late regeneration phases is also useful to compare pathway deregulation across species. We include in the early phase DE genes for the first two time points in *H. magnipapillata*, *S. mediterranea*, and rat liver regeneration, and for the first time point in *A. japonicus*. We perform a gene ontology (GO) analysis of DE genes separately for each organism using the David and Panther databases, annotating the relative Panther families (see Methods section). During the early phase, upregulated genes in the various species are associated with GO biological processes referring to “cell–cell communication”, “DNA repair”, and “initiation of transcription”. We observe a downregulation of metabolic processes, cell adhesion and, interestingly some immune-response-related genes (cell adhesion, receptor-mediated endocytosis, cellular component biogenesis, lipid metabolic process). Upregulated genes are mostly classified, according to GO molecular function categories, as receptors, transmembrane transporters, and transcription factors, while downregulated genes are mostly associated to binding proteins and oxidoreductases. On the other hand, DE genes during the late phase, defined using the last two time points, are more heterogeneous and related to the regeneration process characteristic of each species (e.g. kidney morphogenesis in *A. japonicus*). In the late phase, we find GO terms, such as “cell adhesion”, “central nervous system development”, “development and morphogenesis of epithelium and embryo”, and “extracellular matrix organization”.

We also investigate the pathways related to early and late DE genes, according to KEGG pathway database.^28^ We find large pathways such as “NF-kB signaling pathway”, “Toll-like receptor signaling pathway”, and some human disease-related pathways, such as Alzheimer, Parkinson, and cancer. Further investigation on the role of these DE genes in disease-related pathways, allows us to observe that most of them are membrane proteins or mitochondria-related genes. This suggests that these pathways are simply related to inflammation, which is a common feature of all these diseases. On the other hand, the constantly overexpressed genes are mostly related to defense-response GO terms.

### Common patterns in the regeneration process of different species

**Fig. 3:**
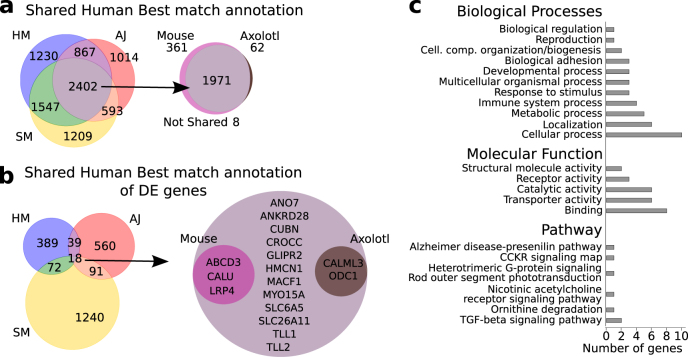
Transcriptome co-annotation. **a** Human best-match of genes co-annotated between *H. Magnipapillata* (HM), *S. Mediterranea* (SM), and *A. Japonicus* (AJ). Most of these genes are also ortholog in mouse and annotated in axolotl. **b** The subset of human genes that are both co-annotated and differentially expressed in all the three considered organisms have a small superimposition with genes differentially expressed during mouse and axolotl regenerations. **c** Panther annotation of relevant biological processes, molecular function, and pathways of the 18 human best-match proteins that are both co-annotated and differentially expressed in *H. Magnipapillata*, *S. Mediterranea*, and *A. Japonicus*

After having analyzed separately individual regenerating species, we now focus our attention on common gene expression patterns. Figure 3a shows that 2402 genes can be co-annotated for all the three species. Furthermore, we find 18 genes that are both co-annotated and DE across the three species (Fig. 3b).

Performing a functional annotation of the DE 18 genes sharing human best match annotation, we obtain a list of interesting functions including “proteases involved in developmental processes” (Toll-like protein 1 and 2), related to “calcium ion binding” (Calmodulin Like 3, Calumenin), “cell junction”, “structure and migration” (Hemicentin-1, GLIPR2, MACF1, MYO15A), and the “cancer-related scramblase ANO7” (Fig. 2b). The analysis of the pathways yields six pathways associated to seven genes: “Alzheimer disease-presenilin” (P00004), “CCKR signaling map2 (P06959), “Heterotrimeric G-protein signaling pathway-rod outer segment phototransduction” (P00028), “Nicotinic acetylcholine receptor signaling” (P00044), “Ornithine degradation” (P02758), and “TGF-*β* signaling” (P00052) (Fig. 2b).

We are interested in assessing the degree of conservation of the genes involved in regeneration processes, not only among the considered organisms but also in more complex organisms with reduced regenerative capabilities, such as vertebrates and mammals. Thus, we repeated the analysis for *A. mexicanum*, obtaining 2033 co-annotated genes, of which 1971 are also annotated in mouse. We also report that 361 genes are co-annotated in all the three species and in mouse, but not in *A. mexicanum*. According to human best-match annotation, two of the 18 co-annotated DE genes in three species considered above (Calmodulin and Ornithine decarboxylase) are also DE during limb regeneration in *A. mexicanum*.

Furthermore, we analyzed 2436 murine and 767 rat DE genes during liver regeneration after PH.^24,25^ Among the murine DE genes reported by Pibiri and coworkers,^25^ 1901 genes have a correspondence in the Swissprot database. Rat DE genes considered by Xu and coworkers^24^ can be mapped to 454 Swissprot entries. Murine data are more complete and are therefore used as reference for DE genes annotation. We searched for the human orthologs of murine DE genes and three of them (ABCD3, CALU, LRP4) are in common between all the organisms (Fig. 3b).

While Swissprot annotation appears to catch the conserved core of DE genes involved in regeneration, a large fraction of the DE genes are not annotated (or have a low-confidence match) in the Swissprot database. Therefore, we investigated the existence of possible correspondence between the DE genes in the various species, even if they are not present in the Swissprot database. Reciprocal alignment of *H. magnipapillata*, *S. mediterranea*, and *A. japonicus* transcriptomes yields 38, 105, and 44 DE genes, respectively, that are putative orthologues among the species. Moreover, we find 31 DE genes in *A. mexicanum* that are putative orthologues to all the other three species. These results are compatible with those obtained using Swissprot alignment.

### Macrophage-related injury response in regeneration

Inflammatory response after injury is not only a defense mechanism against external microbes but also plays a crucial role during regeneration.^9,11,18,29^ In particular, cells with phagocytic capacity represent one of the first barriers against infection and contribute to regeneration by eliminating dead cells at the wound site. Hence, their combined action is essential for a successful regeneration process.^4,30^ In order to verify the presence of specific inflammatory response in the species considered, we look for the presence of macrophage and neutrophil-related genes in our DE gene lists.

First, we combine the list of human and murine macrophage-related genes collected by Gautier and coworkers,^31^ Greaves,^32^ and by the ProteinAtlas project^33^ (see section “Methods” for details). Our datasets share five macrophage-related genes (MARCO, Mrc1, Siglec1, Csf1r, CD14) and six others are in common between at least two groups (MSR1, CD80, CD68, CD11c,TRAP, Adgre1). Direct alignment of *H. magnipapillata*, *S. mediterranea*, and *A. japonicus* transcriptomes against the sequences of the five most represented genes allows us to detect the presence of putative orthologues of MARCO in all the species considered, including mouse and rat. This gene is upregulated in *A. japonicus* and *H. magnipapillata*, and downregulated in *S. mediterranea*, during both head and tail regenerations. The same procedure, repeated on the whole set of genes obtained by Gautier and coworkers,^31^ allows us to detect a larger subset of 21 conserved genes (see Supplementary data 3), which we compare to our list of DE genes for each species. Only four of those genes are detected as DE in mouse (Tgf*β*, Marco, Aldh6a1, and Sgk1) and two of them (Abcc3 and Sgk1) in rat, while in the other species, a few genes (10 for *S. mediterranea*, 5 for *A. japonicus*, 1 for *H. magnipapillata*, and 3 for *A. Mexicanum*) have a human best match annotation on 11 of the 21 macrophage genes.

**Fig. 4:**
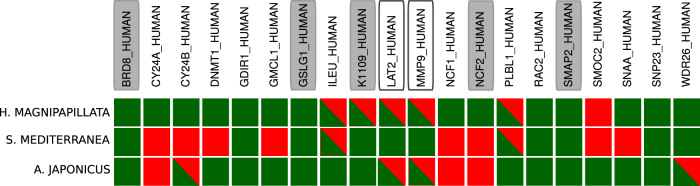
Macrophage-related and neutrophil-related genes. List of the 20 macrophage-related and neutrophil-related proteins present (green square) in the human best match annotation of at least one of the considered organisms. List match annotation of the same genes (half green square) is included for comparison. Filled rectangles indicate DE genes considering only human best match annotation, empty rectangles indicate DE genes when list-match annotation is considered. For a complete list-match annotation, see also Suppl. Figure 4

Next, we extend our search to include human macrophage or neutrophil-related genes in the Swissprot database. As a whole, 20 neutrophil or macrophage-related genes are detectable as human best match in the transcriptome of the three organisms, five of them being DE during regeneration. We notice that the annotation strategy used so far and based on the reference human genome might be too stringent when we analyze distantly related species like those considered here. An alternative strategy is provided by *list-match* annotation, where we associate a gene in each one of the three species with the list of genes, taken from all the possible reference genome, that are aligned with a confidence level smaller than *p* = 10^−15^ (see Supplementary Figure 3 and Methods section). As shown in Fig. 4, list-match annotation could improve the detection of closely related genes in the organisms having different human best-match annotation (e.g. MMP9/MMP8, see also Supplementary Figure 4). Expression pattern of this set of DE genes appear to be conserved within the same organism (Supplementary Figure 5).

### Genes related to the epithelial–mesenchymal transition (EMT)

**Fig. 5:**
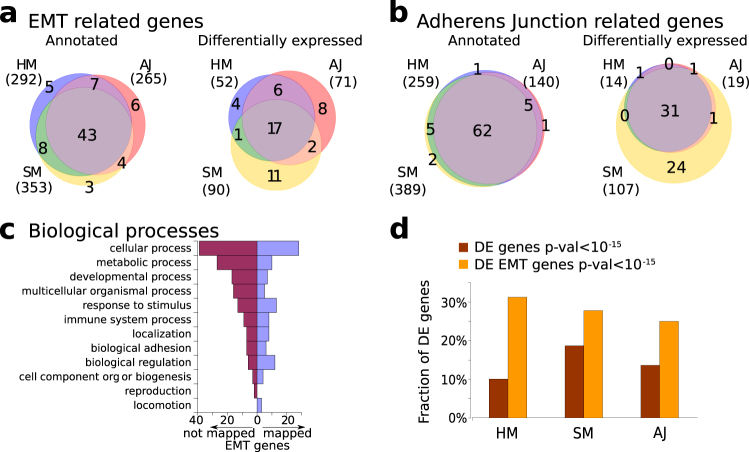
Signature of masenchymal phenotype during regeneration process. Number of genes related to (**a**) EMT and (**b**) involved in adherens junction detected in *H. magnipapillata* (HM), *S. mediterranea* (SM), and *A. japonicus* (AJ) transcriptomes (left) and differentially expressed during regeneration process (right). Numbers reported close to the species label indicates the corresponding number of genes. (**c**) Biological processes annotation of the 76 EMT-related proteins represented in the considered organisms (right, violet bars) and the 54 not detected (left, purple). (**d**) Histogram shows the comparison between fraction of total DE genes and EMT-annotated genes that result in deregulated during regeneration process for the three organisms

Plasticity allows cells to rapidly migrate towards the injury ensuring a fast wound closure and an effective regeneration process.^34^ We thus investigate the presence of well-known EMT markers during regeneration. As described in the the Methods section, we search for 130 human and murine EMT genes in the transcriptomes of the regenerating species. As in the case of macrophage-related and neutrophil-related genes, the application of human best match annotation reveals only a small set of EMT genes (Supplementary Figure 6). Hence, we resort again to list-match annotation to obtain less strict annotation. Using this method we find that about two-thirds of EMT genes are expressed and DE, even if the core of common deregulated genes is limited (Fig. 5a). We also consider human and zebrafish genes involved in the “Adherens Junction” pathway (see section “Methods”), since a deregulation of this pathway is related to EMT. Using the same strategy described above, we selected 89 Swissprot-annotated genes from this pathway and found a large core of representative genes strictly conserved in the considered organisms and DE during all the regeneration processes (Fig. 5b). Figure 5c reports the biological processes associated to the EMT genes considered here. Finally, we compare the fraction of total annotated DE genes and EMT-annotated genes for the three species (Fig. 5d).

## Discussion

The main goal of the present paper is to identify genes that are DE in similar ways during regeneration across distantly related species, to see if any common feature is still present across all species. To this end, we perform a curated annotation of available time-dependent transcriptomes. Mapping the different transcriptomes on a common genome allows us to detect conserved patterns during the early and late regeration phases. DE genes are found to be more likely to be shared across different species during the early regeneration phase. On the other hand during late regeneration, DE genes are related to the characteristics of the specific tissue or organism considered.

In principle, every organism maintains some capability to regenerate, but while invertebrates can regenerate complex structures and even the whole body, among vertebrates, amphibians are the only animals that can regenerate anatomically complete and fully functional tissues and organs. Mammals have the regenerative capacity to restore the function of the liver post PH, but recover only the organ mass and not its anatomy. Indeed, the early phase of PH displays similarities with the gene expression pattern observed during the regeneration of the three distant species we considered here. This suggests that there is a remembrance of a primordial regeneration capacity that is common to all organisms, including mammals. The data also suggest that the machinery is still there and, even if the genes are different, pathways and functions involved are similar. A recent paper has shown that a small injury in axolotl is sufficient to restart the regeneration program of a previously amputated limb.^35^ This observation is in agreement with our data showing that there is an imprint of regeneration during the very first response to injury.

It has been suggested that the regenerative capacity lost in mammals during evolution has been replaced by the presence of a complex immune response acting during tissue repair.^17^ Failure of organ regeneration is often associated with scarring or fibrosis due to the inflammatory response produced at the injured site. On the other hand, a recent paper shows that in PU.1 null mice the deletion of a transcription factor, which is required to produce specific hematopoietic lineages yields the absence of both macrophages and neutrophils.^30^ In these mice, skin wounds were not only repaired at the same rate as wounds in wild-type siblings, but this happens without inflammation or scar formation, while the complete ablation of the sole macrophages cells affects regeneration.^18,29^ These data support the idea that a combined action of macrophages and neutrophils in inducing and controlling inflammation is necessary for an efficient tissue repair. In agreement with this evidence, we show a signature of neutrophil or macrophage-like cells in the early inflammatory response during the regeneration process of *H. magnipapillata*, *A. japonicus*, and *S. mediterranea*. Intriguingly, early downregulation of macrophage mannose receptor 1 (Mrc1) can be attributed to the presence of pro-inflammatory macrophages during this phase,^4^ while Mrc1 seems to be expressed preferentially in macrophages that are likely to appear during the post-inflammatory phase.^36,37^ Our findings show that in these distantly related species there is already a signature that is associated to those cells. This suggests that macrophages and neutrophils are the result of the evolution of a primordial function.

Another interesting result is the identification of a signature associated to EMT in the regeneration process of *H. magnipapillata*, *A. japonicus*, and *S. mediterranea*. It is well known that tissue regeneration in mammals involves the EMT of cells near the injured site. This result strengthens the idea that the complex tissue repair strategy in mammals is related to the regeneration capacity present in those distant species.

All together, our strategy helps uncover the presence of a set of genes whose expression and function are critical among all species during early regeneration, suggesting the importance of these genes for their survival. It is tempting to speculate that inhibiting these genes could be useful to treat pathologies due to excessive fibrosis in humans. On the other hand, further investigation of non-conserved genes, whose expression was lost in vertebrate during evolution could be useful for future regeneration therapies.

## Methods

### RTs and annotation

We considered *H. magnipapillata*^19^ (last updated January 2016), *S. mediterranea*,^20^ and *A. japonicus*^21^ assembled transcriptomes and the corresponding data from different regeneration experiments. Briefly, RT of *H. magnipalliata* was obtained immediately after head amputation and from regenerating head after 0.5, 3, 6, 12, 24, 48 h; *S. mediterranea* data refer to both head and tail regenerations and are obtained 6, 12, 24, 36, 48 and 72 h after amputation, as well as from unharmed animals; *A. japonicus* individuals were subject to intestine removal and data on regenerating tissue were collected 3, 7, 14 and 21 days after evisceration. Transcriptome from axolotl *A. mexicanum* limb regeneration^23^ comprises 12 time points, up to 21 days after injury. In order to improve the annotation process, transcripts are clustered using CD-Hit^38^ with a similarity thresholds between the sequences of 80%. We refer to these clustered transcripts as genes. The Swissprot/Uniprot database^26^ (last accessed 5 July 2016) and NCBI NR sequences database (last accessed 20 May 2016^27^) were used as input for Blast+^39^ (ncbi-blast version 2.31) to annotate all the considered transcriptomes using a very permissive *p*-value threshold of 10^−2^. *p*-Values are corrected for multiple alignments according to the algorithm^39^ using fixed database size (-dbsize 1.5 × 10^8^).

We discarded from the alignments all the hits to non-eukaryotic sequences, according to Uniprot and NCBI taxonomy classifications, and the BLAST hits having a *p-*value > 10^−15^. For each gene, the human hit (if existing) having the best average alignment score and lowest average *p*-value over all the corresponding transcripts is the *Human best match*. Furthermore, for each gene, the whole set of Swissprot hits with *p*-value ≤ 10^−15^ was used to define a broader shared annotation between the different organisms. We refer to this annotation as *list-match* annotation (see Supplementary Figure 3).

Human best-match annotation implies that two genes, even if very similar, could be associated to slightly different Swissprot entries, depending on the specific *p*-value of each annotation. List-match annotation allows us to increase the superimposition of co-annotated genes and include more information. Using this criterion, two genes are considered co-annotated if they share more than the 80% of their list-match annotation. Thus, while list-match annotation allows us to neglect small differences in the alignments that could lead to slightly different *p*-values, the method involves an ambiguous match between the genes within and between organisms (e.g. a single *H. magnipapillata* gene could be co-annotated to two *S. mediterranea* genes, sharing with them different hits). Overall, the number of genes resulting co-annotated between the organisms is of the same order of magnitude considering both human best-matches and list-matches, ranging roughly between 2000 and 3000 genes (Fig. 3a and Supplementary Figure 3).

Alignment of the reference transcriptomes of *H. magnipapillata*, *S. mediterranea*, *A. japonicus*, and *A. mexicanum* to the Swissprot database according to this procedure allows us to give a putative annotation to about one-third of the considered genes.

We verified that the distribution of the number of hits is coherent between the species, ranging from one up to hundreds of possible putative annotation, with an average value of $\approx 15$. Reciprocal alignment of the transcriptome was performed using Lastz software^40^ in order to detect similarity between sequences regardless their annotation in available databases. Different thresholds were used for significant matches (30–50% of coverage and 50–70% of alignment of the query sequence) and results were converted to genes.

Microarray gene expression data of regenerating mouse and rat liver after PH experiments were obtained from the works of Pibiri et al.^25^ and Xu et al.^24^. In mouse experiments, 10 weeks and 18 months old animals were sacrificed before and 3, 6, 12, 24, and 36 h after PH. We consider all expression data from both young and old animals. Rat data refer to 11 different time points, from 0 h up to 7 days post injury,^24^ and a selection of 767 well-annotated genes was considered for data analysis, according to the original work. The resulting 2463 murine genes detected as DE during mouse liver regeneration and 767 rat genes were mapped to the Swissprot database, obtaining 1901 and 454 genes, respectively.

### DE genes

Time-course data of RTs were analyzed to detect DE genes, assigning to each gene the total raw count of the corresponding transcripts. Differential expression analysis was performed using edgeR^41^ (R package version 3.12.1), considering only genes with more than 20 raw counts (or 1 rpkm when available) in at least one time point implementing the Benjamini–Hochberg correction. For each time point, we compute the $\mathop {{{\rm log}}}\nolimits_2$ of the gene expression fold change (log_2_*FC*) with respect to the initial time point. Genes with |log_2_*FC*| > 2 and corrected *p*-value < 0.05 were considered as putative DE genes in *S. mediterranea*, *A. japonicus*, and *A. mexicanum*. *H. magnipalliata* genes are considered DE when |log_2_*FC*| > 0.5 and corrected *p*-value < 0.05. When not explicitly specified, we refer to DE genes during both head and tail regeneration processes as *S. mediterranea* DE genes.

### Functional analysis

Gene Ontology analysis was performed on DE genes using the David^42^ (version 6.8) and Panther classification systems^43^ (versions 11.1 and 12.0). Clustering of DE genes according to their global change in expression was performed using both Python-Scipy^44^ (version 0.17.1) and Cluster 3.0.^45^

### Macrophage and neutrophil-related genes

A dataset of 296 human marcophages-related genes were obtained from Protein Atlas Project (http://www.proteinatlas.org,^33^ last accessed October 2016). Murine marcophages-related genes described by Gautier and coworkers^31^ consist of 363 genes overexpressed or exclusively expressed by macrophages in at least two different tissues and between these 14 were exclusively expressed in macrophages in four different murine tissues. Additionally, we considered a set of 16 genes related to different macrophages cells as described by Greaves and coworkers.^32^ CDNAs sequences of these genes and a list of the murine orthologs of human macrophage-related genes were obtained by Ensembl BioMart tool^46^ (last accessed October 2016, Ensembl version 86). All the sequences were searched in the analyzed transcriptomes using Lastz software.^40^ Finally, the Swissprot database was searched for macrophage and neutrophil genes, yielding a list of 153 genes of different species, and 90 of them result in human or can be annotated to a human ortholog.

### EMT signature

A total of 12 gene expression datasets of human and murine cells with epithelial and mesenchymal/ameboid phenotype were downloaded from the GEO-NCBI repository (last accessed May 2017). Seven datasets (GSE14773, GSE17708,GSE20247,GSE23952, GSE79235, GSE82293, and GSE10196) involve human cells subject to EMT due TGF*β*, Snail, or Yap differential expression. Epithelial to ameboid transition is obtained via EphA2, Ilomasta, and Rho (GSE52246). The four experiments considered on murine cells (GSE81033, GSE87472, GSE49073, and GSE50002) include overexpression of Twist and TGF*β*, as well as spontaneous tumoral subpopulation with different phenotypes. Some of these datasets include also mutant cell lines and induced spheroids. For both human and murine data, we investigate differential gene expression between cells with epithelial-like and mesenchymal-like phenotype using R limma package^47^ (log*FC* > 2, adjusted *p*-value < 0.05). Two murine and two human datasets have low differential expression or high variability, resulting in a lack of DE genes. For these datasets, we considered as DE genes those passing a more stringent test (log*FC* > 1, *p*-value < 10^−3^). We considered as EMT-related genes those found DE in at least two out of four murine datasets and three out of eight human datasets obtaining a list of 101 murine and 47 human genes. These can be mapped to 130 genes in the Swissprot database, allowing comparison with our Swissprot annotation of transcriptomes. The list of human and Zebrafish genes involved in the “Adherens junction” pathway was downloaded from the Kegg Pathways database^28^ (last accessed May 2017, release 81.0). These contains, respectively, 72 and 97 genes, mapped on 72 and 17 Swissprot entries. The combination of these two lists was used to search the transcriptomes.

### Code availability

A repository with code used to generate the results of this paper is available at https://github.com/ComplexityBiosystems/Regeneration.

### Data availability statement

All the data analyzed in this paper have been downloaded from publicly available databases as detailed in the Method section.

## Electronic supplementary material

Supplemenrary Information

Dataset S1

Dataset S2

Dataset S3

## Electronic supplementary material

**Supplementary information** accompanies the paper on the *npj Systems Biology and Applications* website (10.1038/s41540-017-0042-z).
