# Supplementary material for: Regeneration in distantly related species: common strategies and pathways
Source: NPJ Syst Biol Appl. 2018 Jan 11;4:5. doi: 10.1038/s41540-017-0042-z (PMC5764997; doi:10.1038/s41540-017-0042-z)
Supplement: Supplementary file 1 — Supplemenrary Information [file 41540_2017_42_MOESM1_ESM.pdf]

Regeneration in distantly related species: common strategies and pathways

Supplementary Figures

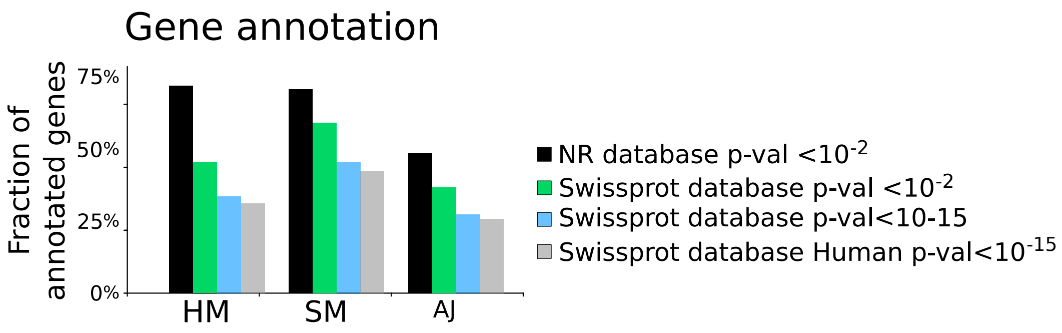

Supplementary Figure 1: **Comparison between different databases.** The histogram represents the frac- tion of *H. magnipapillata* (HM), *S. mediterranea* (SM) and *A. japonicus* (AJ) genes having at least one hit in NCBI non-redundant protein (NR) or Swissprot database with different p-value thresholds as in legend. While *S. mediterranea* globally has a larger fraction of annotated genes, the trend is shared between the three organisms.

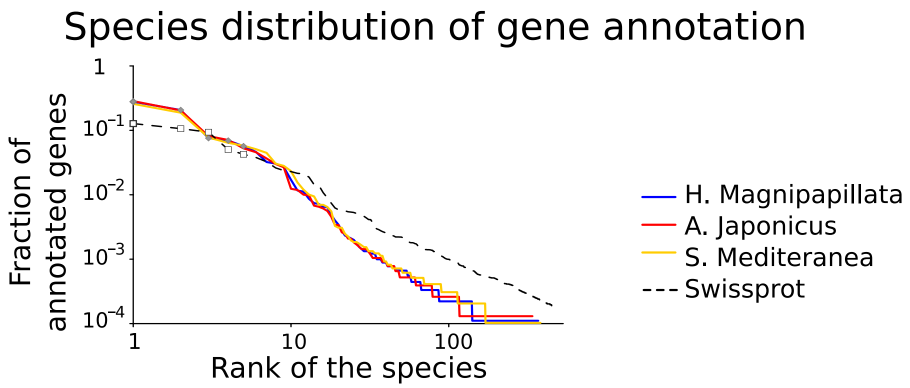

Supplementary Figure 2: **Matching with different species.** The graph shows, for each considered organ- ism, the ranked fraction of genes having the lowest p-value annotation on Swissprot database on different species. The two most frequent species are Human and mouse for all the three organisms. Successive most-frequent species for *A. japonicus* and *H. magnipapillata* are *Bos taurus*, Rat, *Danio rerio*,*Xenopus*, Chicken and *D. melanogaster*. These same organisms are the most represented in *S.mediterranea*, with a different order. A similar hierarchy is present in the Swissprot database (black dashed line), and the large differences between the distributions are essentially due to the presence of *A. thaliana*, and Yeast (*S. cerevisiae* and *S. pombe*) in the top annotated species.

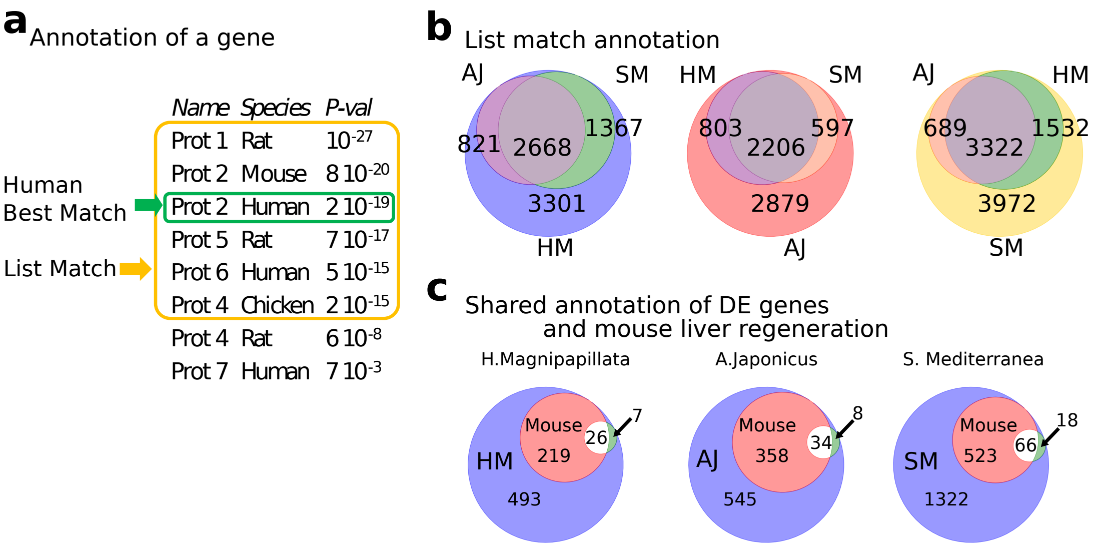

Supplementary Figure 3: **Transcriptome annotation.** (a) Illustration of the two methods used to annotate genes on the Swissprot database. The diagrams represent the number of genes (b) and DE genes (c) having list-match annotation on the Swissprot database for *H. magnipapillata* (HM), *S. mediterranea* (SM) and *A. japonicus* (AJ). We consider genes sharing at least 80% of their Swissprot annotation with genes of the other two species as co-annotated. For DE genes, the subset of genes co-annotated on differentially expressed in mouse (red) and the other two species (green) are also indicated. The subset of DE and co-annotated genes in the four species (white) is significantly larger than in the case of human best match annotation. As a whole, they correspond to 87 different human best-matches, and only three of these are shared between *H. magnipapillata*, *S. mediterranea* and *A. japonicus*.

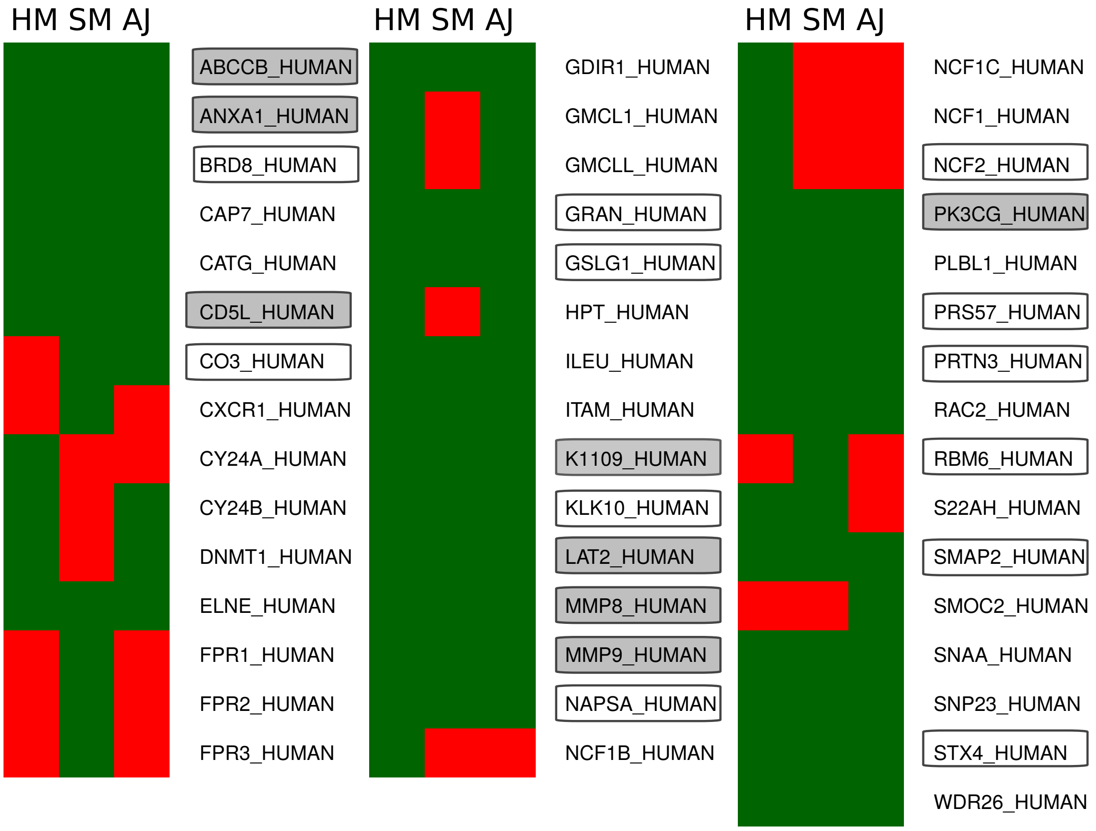

Supplementary Figure 4: **Macrophage and Neutrophil related genes.** Complete list of the 47 macrophage and neutrophil related genes human list-match annotated in at least one of the three considered species. For each considered organism, human proteins are indicated in green if present, while red indicates the absence from that specific organism. Filled rectangle indicate genes differentially expressed in at least two species, empty rectangles indicate DE genes in one specie.

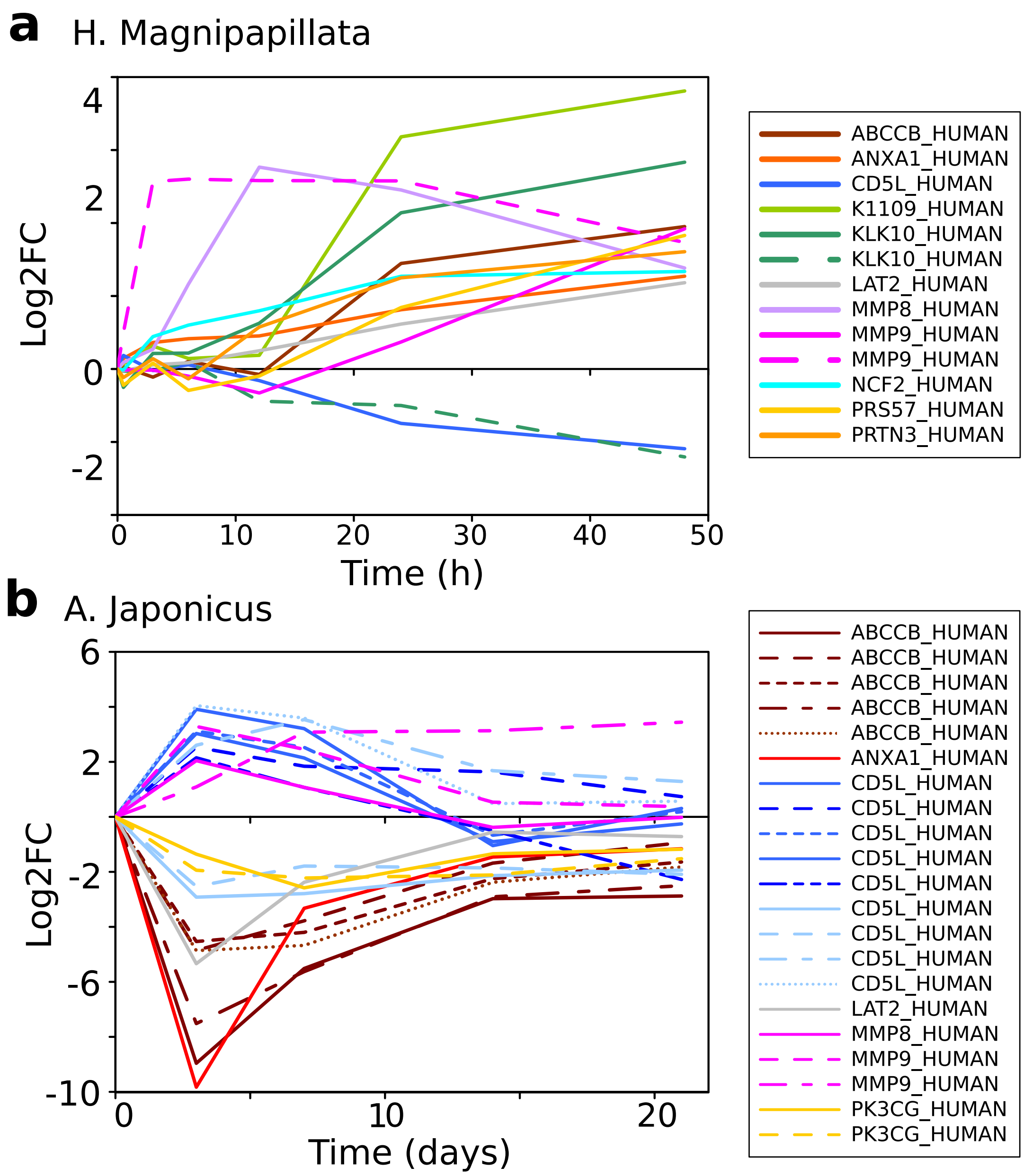

Supplementary Figure 5: **Expression of macrophage and neutrophil related genes.** Figure shows expression of the *H. magnipapillata* (a) and *A. japonicus* (b) genes that are differentailly expressed and annotated on macrophage or neutrophil related genes.

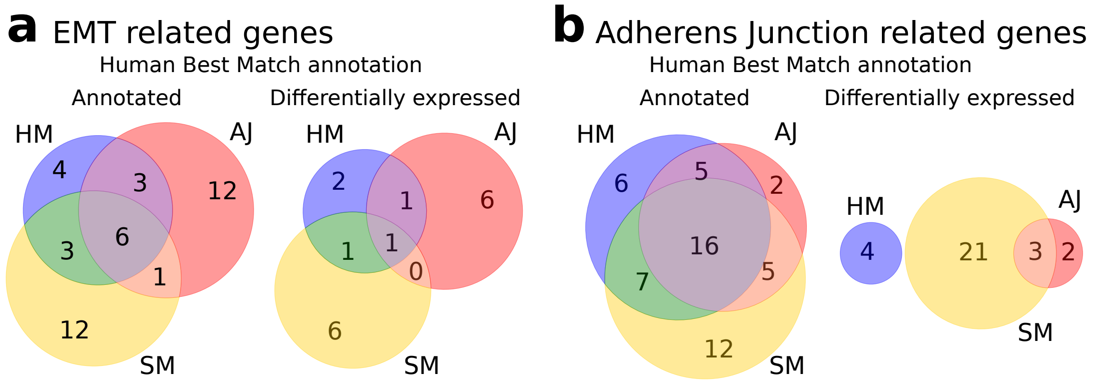

Supplementary Figure 6: **Signature of masenchymal phenotype during regeneration process.** Number of human and murine ortholog genes related to EMT (a) and human genes involved in adherens junction (b) detected using human best match annotation in *H. magnipapillata* (HM), *S. mediterranea* (SM) and *A. japonicus* (AJ) transcriptomes (left) and differentially expressed during regeneration process (right). In order to maximise the shared subest of genes, the reference list of 130 human and murine EMT related genes used in Fig.5 is converted to 126 human genes, while only the 72 human genes were considered for adherens junction annotation.
